# MicroRNA-155 Suppresses Mesangial Cell Proliferation and TGF-β1 Production *via* Inhibiting CXCR5-ERK Signaling Pathway in Lupus Nephritis

**DOI:** 10.1007/s10753-018-0889-1

**Published:** 2018-09-12

**Authors:** Jie Kong, Liuxia Li, Zhimin Lu, Jiamin Song, Jiaxin Yan, Junling Yang, Zhifeng Gu, Zhanyun Da

**Affiliations:** 1 Department of Rheumatology, Affiliated Hospital of Nantong University, No. 20, XiSi Road, Nantong, 226001 Jiangsu Province People’s Republic of China; 2 Research Center of Clinical Medicine, Affiliated Hospital of Nantong University, No. 20, XiSi Road, Nantong, 226001 Jiangsu Province People’s Republic of China

**Keywords:** CXCL13, miR-155, mesangial cell, lupus nephritis

## Abstract

Increasing evidence shows miR-155 plays an important role in regulating inflammatory processes in systemic lupus erythematosus (SLE), especially in lupus nephritis (LN). Because the chemokine CXCL13 is implicated in the pathogenesis of LN, here we examined whether miR-155 can modulate the activity of CXCL13 or its receptor CXCR5. We determined the expression of CXCL13 in normal and MRL/lpr mice and found elevated levels of CXCL13 in the kidneys of MRL/lpr mice compared with normal kidneys. Besides, CXCL13 expression was mainly detected in the glomerulus, specifically to mesangial areas. We then transfected a miR-155 mimic in human renal mesangial cells (HRMCs) to overexpress miR-155 and detected decreased protein levels of CXCR5 by western blot analysis. Transfection of the miR-155 mimic into CXCL13-treated HRMCs resulted in a significantly reduced proliferation rate of HRMCs as measured by the cell-counting assay and flow cytometry. Moreover, increased intracellular miR-155 also led to decreased phosphorylation of ERK and TGF-β1 production. Together, these results revealed that miR-155 may play a role in the pathogenesis of LN.

## INTRODUCTION

Systemic lupus erythematosus (SLE) is an autoimmune disease with immune complexes formation and deposition in multiple organs, among which, the kidney is one of the major target organs [12, 34]. At least 30 to 60% SLE patients have lupus nephritis (LN), and almost all patients have pathological kidney involvement [2, 6]. Notably, 10% of LN patients progress to end-stage renal disease (ESRD) [16]. Consequently, LN is one of the leading causes of death in SLE [7, 26]. In recent years, early diagnosis, standardized treatment, and new immunosuppressive agents such as mycophenolate mofetil [19], anti-CD20 monoclonal antibody, belimumab [11], and other drugs have significantly improved the prognosis of LN. However, the mortality rate within 1–5 years in severe refractory LN patients is still high [1]. Therefore, it is of great importance to explore the pathogenesis of LN and search for new therapeutic targets.

MicroRNAs (miRNAs) are newly discovered non-coding RNA molecules composed of ~ 22 nucleotides, and they can regulate the expression of target genes by binding to the 3′ untranslated region of the mRNA (3′ UTR) [31]. MiRNAs play important roles in various physiological and pathological processes such as cell proliferation, differentiation, and apoptosis by downregulating the expression of target genes [4, 15]. MiR-155 is a member of the miRNA superfamily that mediates innate and adaptive immune responses and plays an important role in regulating blood cell generation, inflammation, and immune responses [18]. Previous studies used bioinformatic analysis for miRNA target prediction of genes and found that miR-155 was involved in the ERK/MAPK signaling pathway [23]. Moreover, miR-155 has been reported to inhibit the secretion of inflammatory factors by downregulating the ERK/MAPK signaling pathway [3]. Recent studies found that the serum levels of miR-155 were significantly lower in SLE patients than those in healthy individuals. Thus, miR-155 may serve as an additional serological marker for SLE [32].

Here, we examined the effects of increased intracellular levels of miR-155 activity on the proliferation of human renal mesangial cells (HRMCs) and the expression of the CXCR5, which were inhibited by the overexpression of miR-155. Moreover, both protein levels of p-ERK and transforming growth factor β 1(TGF-β1) production were also reduced in miR-155-overexpressed HRMCs. Therefore, our results suggest that miR-155 can suppress CXCL13-induced proliferation of HRMCs in LN by downregulating the CXCR5-ERK signaling pathway.

## MATERIALS AND METHODS

### Cell Culture

HRMCs were obtained from JENNIO Biological Technology and cultured in RPMI 1640 medium supplemented with 10% fetal bovine serum (FBS) and 1% penicillin/streptomycin, at 37 °C in a 5% CO_2_-humidified atmosphere.

### Cell Transfection

HRMCs were placed into 6-well plates at 37 °C under 5% CO_2_ for 24 h before transfection. The miR-155 mimic (Biomics Biotech, China) was transfected into cells using Lipofectamine 2000 (Invitrogen by Life Technologies, USA) at 50% confluency. We used siRNA (Biomics Biotech, China) to silence the expression of CXCR5 in HRMCs. The optimal concentration for transfection was established at 100 nmol/mL siRNA. The transfection medium contained no FBS or penicillin/streptomycin. After 4–6 h of transfection, the medium was replaced by RPMI 1640 with 10% FBS, and the cells were then incubated at 37 °C with 5% CO_2_ for 48–72 h. Total protein was collected.

### Cell Proliferation Assay

Cell proliferation was monitored using the Cell Counting Kit-8 (Sangon Biotech, Shanghai, China) according to the manufacturer’s instructions. Aliquots of 100 μL cell suspension were plated into 96-well plates at 1 × 10^3^ cells per well and cultured in the growth medium for 24 h. Cells were then treated with 0.5 ng/mL recombinant human CXCL13 (R&D Systems, USA). HRMCs were transfected with either 50 nmol/mL miR-155 mimic or 100 nmol/mL siRNA in RPMI 1640 (without serum or antibiotics). The number of viable cells was assessed at 0, 6, 12, 24, and 36 h after treatment. Each well was added with 10 μL CCK-8 solution, incubated for 2.5 h in the dark, and measured the absorbance at 450 nm using a microplate reader (BioTek, USA).

### Flow Cytometric Analysis

Cell cycles of HRMCs with CXCL13 treatment or transfection with siRNA or miRNA were determined by flow cytometry. Cells were digested in 500 μL 0.05% trypsin-EDTA for 5–7 min and added 500 μL RPMI 1640 supplemented with 10% FBS to inactivate trypsin. Cells were then centrifuged at 1200 rpm for 5 min, washed and resuspended in cold PBS twice, and incubated at − 20 °C in 70% ethanol for at least 24 h. They were then permeabilized with 200 μL PBS containing 1% Triton X-100 for 10 min. Finally, the cells were resuspended in 500 μL propidium iodide (PI)/RNase staining buffer (BD Pharmingen, USA) for 15 min in the dark and analyzed by flow cytometry. MFLT32 Soft was used to calculate the fraction of cells in S phase.

### Immunohistochemistry

For immunohistochemistry, kidney samples were fixed in 4% buffered paraformaldehyde and embedded in paraffin. This was followed by deparaffinizing the samples twice with xylene for 15 min and rehydrating in descending grades of alcohol (100–70%). Then, sections were heated in a microwave oven at 100 °C with 10 mM citrate buffer (pH 6.0) for 10 min to retrieve the antigen and washed three times with PBS. Endogenous peroxidase was inhibited by incubation in 3% H_2_O_2_ for 10 min. Samples were then incubated with anti-CXCL13 (Abcam, USA) as primary antibody at 4 °C overnight, followed by a subsequent incubation with peroxidase-labeled goat anti-rabbit lgG for 20 min at room temperature. Peroxidase activity was detected using diaminobenzidine (DAB) as substrate, and the nuclei were counterstained with hematoxylin. MRL/MPJ mice were used as control. The study was approved by the Ethics Committees of Affiliated Hospital of Nantong University. The approval number is 2017-L096.

### Immunofluorescent Staining of Kidney Tissue

The expression and localization of CXCL13 in renal tissues were determined by immunofluorescence assay. In brief, kidney samples were deparaffinized, rehydrated, and antigen-retrieved as described. Double-staining was achieved by incubating the specimen with anti-CXCL13 (Abcam, USA) and anti-collagen 4 (Abcam, USA) as primary antibodies at 4 °C overnight, followed by incubation with daylight594 goat anti-rabbit IgG (Abbkine, USA) and daylight488 goat anti-mouse IgG (Abbkine, USA) as secondary antibodies at room temperature for 2 h in the dark. After three washes with 1 × PBS in the dark, the samples were mounted with 4′,6-diamidino-2-phenylindole mounting medium (DAPI, Beyotime Biotechnology, China) for 10 min. Images were captured using an immunofluorescence microscope (Olympus, USA).

### Immunofluorescent Staining in Cell Culture

Cells were first fixed in 4% paraformaldehyde for 40 min, washed three times with PBS, and then permeabilized with 1% TritonX-100 (Beyotime Biotechnology, China) at 4 °C for 10 min, before incubating with primary antibody anti-phospho-p44/42 MAPK(ERK1/2) (Cell Signaling Technology, USA), at 4 °C overnight. After washing with 1 × PBS, cells were incubated with daylight594 goat anti-rabbit IgG (Abbkine, USA) for 2 h in the dark. The nuclei were stained with DAPI (Beyotime Biotechnology, China). Thereafter, the coverslips were viewed under an immunofluorescence microscope (Olympus, USA).

### Enzyme-Linked Immunosorbent Assay

The concentrations of TGF-β1, MCP-1, and IL-1 in the cell culture supernatants were measured using an ELISA kit (R&D Systems, USA) according to the directions of the manufacturer.

### Western Blot

Cells were lysed in 5 × SDS-PAGE Sample Loading Buffer, 100 mM RIPA Lysis Buffer, and 1 mM PMSF (Beyotime Biotechnology, China) and subsequently heated to 95 °C for 5 min. Total protein was quantified by BCA (Beyotime Biotechnology, China). The proteins were transferred to a polyvinylidene difluoride membrane (PVDF) using a Mini Trans-Blot apparatus (Bio-Rad, Hercules, USA). The filters were incubated in TBST with 5% nonfat dry milk for 1–2 h at room temperature and then at 4 °C overnight with anti-CXCR5 (Abcam, USA), and anti-phospho-p44/42 MAPK(ERK1/2) (Cell Signaling Technology, USA) or anti-p44/42 MAPK(ERK1/2) (Cell Signaling Technology, USA) and GAPDH antibody (Proteintech, USA) and then washed with TBST. After further incubation with secondary antibody conjugated with horseradish peroxidase (HRP) for 1 h at room temperature, relative expression levels of protein were quantified using Quantity One software by ECL.

### Statistical Analysis

Data were collected from three independent experiments and shown as the means ± standard deviation (SD). Statistical analysis was performed using GraphPad Prism. Oneway analysis of variance (ANOVA) was used to indicate the differences. *p* < 0.05 was defined as significant.

## RESULTS

### CXCL13 Is Highly Expressed in the Glomerular of LN Patients

**Fig. 1:**
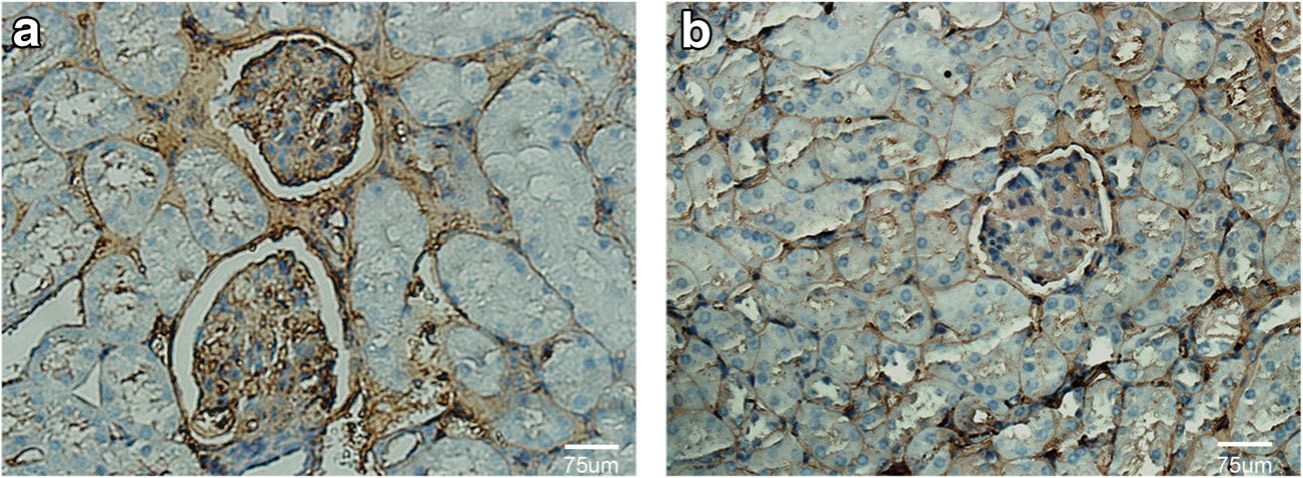
Detection of CXCL13 in mouse kidney tissues using immunochemical staining. Staining of CXCL13 in MRL/lpr mice (**a**) and control (**b**). Original magnification, × 400.

**Fig. 2:**
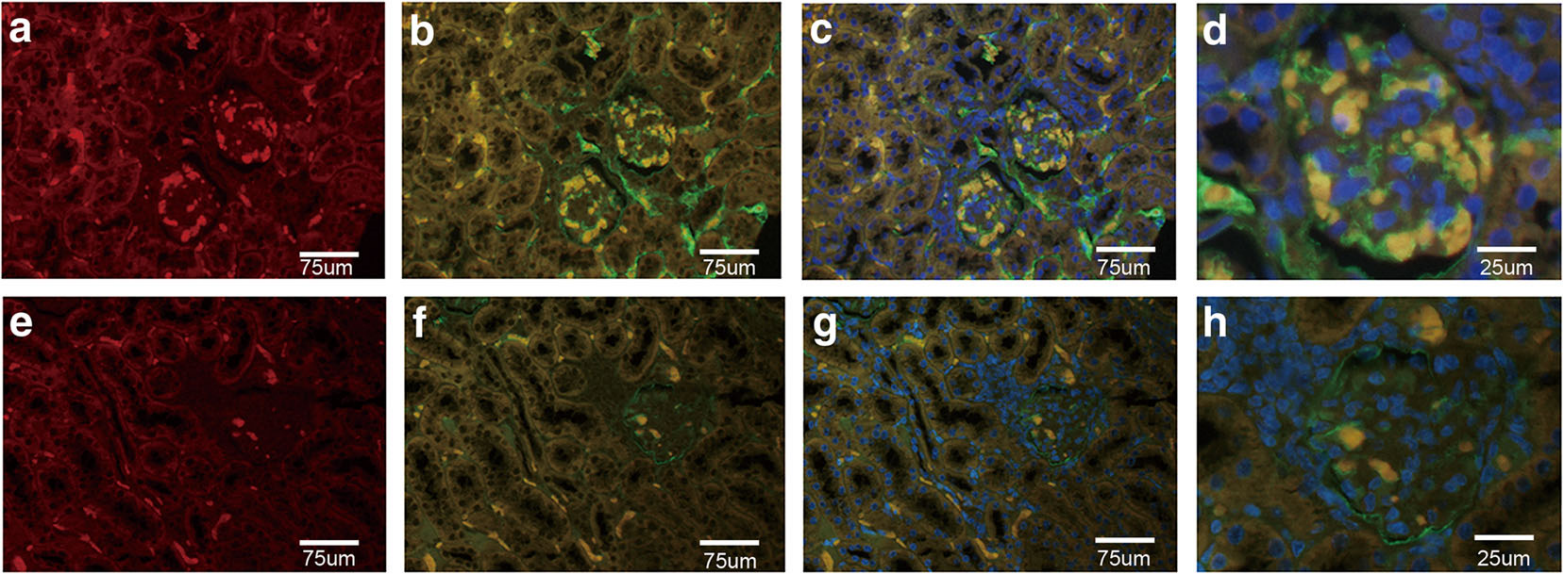
Detection of CXCL13 and collagen 4 expression in renal tissues by immunofluorescent microscopy. Colocalization of CXCL13 (red) and collagen 4 (green) merged to yellow in images. (**a**–**d**) Low expression of CXCL13 in glomerulus, renal tubules, and renal interstitium in control mice. (**e**–**h**) High expression of CXCL13 in glomerulus of MRL/lpr mice. (**d**, **h**) The indicated areas in Figs. C and D showing the location of CXCL13 and collagen 4 in the glomerulus. Original magnification, × 400.

The B lymphocyte chemoattractant BLC/CXCL13 is a CXC chemokine which involved in kidney pathogenesis of LN [10]. Our previous study indicated that the serum concentrations of CXCL13 were significantly higher in LN patients than those in healthy controls [5]. To determine whether the expression of CXCL13 is upregulated in the renal tissue of LN patients, we detected the level of CXCL13 in paraffin-embedded kidney tissues from normal and MRL/lpr by immunohistochemisty and immunofluorescent (CXCL13 and collagen 4) staining. We found that the expression levels of CXCL13 in the kidneys of MRL/lpr mice were significantly increased in comparison to normal mice (Fig. 1 and Fig. 2). Moreover, CXCL13 was located mainly in glomerular regions; however, the staining of CXCL13 was much weaker in renal tubule and renal interstitium regions (Fig. 2e–h). The colocalization of CXCL13 (red stain) and collagen 4 (green stain) was observed in the mesangial areas (Fig. 2d, h). Thus, there is a possibility that the mesangial cells are responsible for the high expression of CXCL13.

### Increasing the Abundance of miR-155 Reduces CXCL13-Induced Proliferation of HRMCs

The level of miR-155 in the serum of SLE patients was significantly lower than that of healthy individuals [32], suggesting a protective role of miR-155 in reducing the proliferation of mesangial cells, endothelial cells, or podocytes in LN patients. We raised the abundance of miR-155 in HRMCs by transfection with a miR-155 mimic and then used the CCK8 kit to examine the effects of the elevated miR-155 on the growth of the cells. The HRMCs transfected with a miR-155 mimic or transfected with siRNA to silence CXCR5 were then treated with or without CXCL13, and cell variability was then detected after 0, 6, 12, 24, and 36 h.

**Fig. 3:**
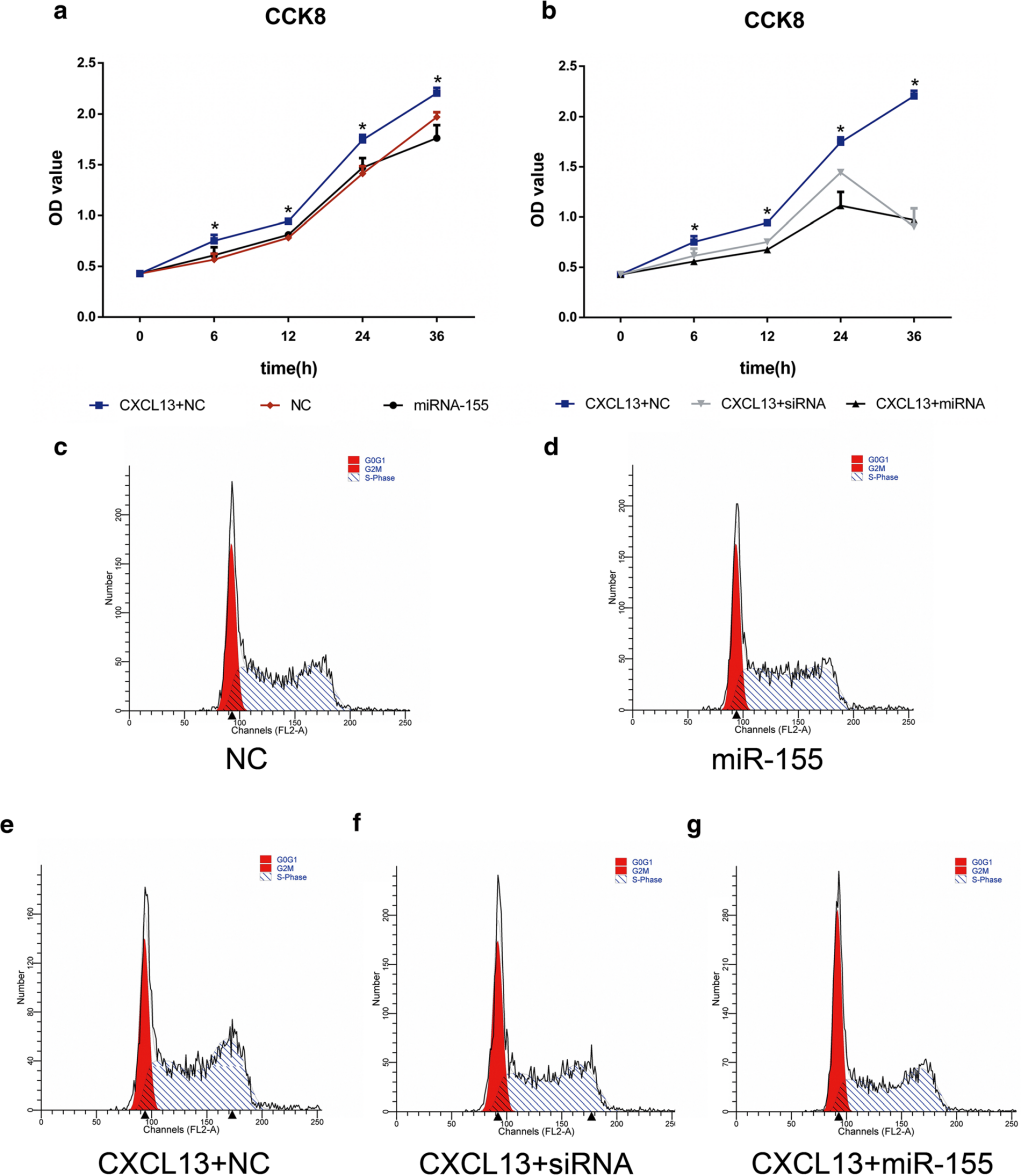
Effect of transfection with a miR-155 mimic on the proliferation rate of HRMCs stimulated by CXCL13: analysis with the CCK8 kit and flow cytometry. Optimal concentrations of CXCL13 (0.5 ng/mL), miR-155 mimic (50 nmol/mL), and siRNA for the silencing of CXCR5 (100 nmol/mL), determined previously, were used in the transfection experiments. (**a**) Proliferation of CXCL13-treated negative control (NC) (blue line); NC (black line); or miR-155 mimic-transfected cells (red line). (**b**) Proliferation of NC treated with 0.5 ng/mL CXCL13 (blue line); or CXCL13-treated cells transfected with siRNA (gray line); or CXCL13-treated cells transfected with a miR-155 mimic (black line). (**c**–**g**) Fractions of cells in S phase in HRMCs from the above five groups were analyzed by flow cytometry. Statistical analyses were performed by ANOVA followed by Dunnett’s test. *p < 0.05.

We observed that CXCL13 treatment could significantly increase the cell proliferation in HRMCs (Fig. 3a); however, overexpression of miR-155 could significantly abrogate CXCL13-stimulated cell proliferation. No significant difference in the growth rates was observed between non-treated cells and miR-155 mimic-treated cells (Fig. 3a). Moreover, transfection of siRNA to silence CXCR5 could also reverse CXCL13-stimulated HMRC proliferation (Fig. 3b). The results led us to hypothesize that miR-155 may decrease the ability of CXCL13-treated HRMCs to proliferate by downregulating CXCR5.

Flow cytometric analysis was used to determine the fraction of cells in S phase (Fig. 3c–g) during cell proliferation. Consistently, we found that CXCL13 could significantly increase the percentage of cells in S phase compared with non-treated controls (76.09 ± 1.44 vs. 68.88 ± 0.70) (Fig. 3e, c). Increasing the abundance of miR-155 in CXCL13-treated HRMCs reduced the percentage of S phase from 76.09 ± 1.44 to 62.03 ± 0.3, which was similar to that observed after CXCR5-silencing (65.46 ± 0.05), see Fig. 3(e–g) and Table 1. No significant difference in the growth rates was observed between non-treated cells and miR-155 mimic-treated cells (Fig. 3c, d). Briefly, the results of the CCK8 tests and flow cytometric analysis showed that miR-155 could reduce the proliferation of HRMCs by downregulating CXCR5.

**Table 1:** Ratio of HRMCs in S Phase From Different Groups

	S phase (mean ± SD)	*p
NC%	68.88 ± 0.70	–
MiR-155%	68.10 ± 1.57	> 0.05 (*versus* NC%)
CXCL13 + NC%	76.09 ± 1.44	< 0.05 (*versus* NC%)
CXCL13 + MiR-155%	62.03 ± 0.30	< 0.05 (*versus* CXCL13 + NC%)
CXCL13 + siRNA%	65.46 ± 0.05	< 0.05 (*versus* CXCL13 + NC%)

Statistical analyses were performed by ANOVA followed by Dunnett’s test. *p < 0.05 was considered significant

### MiR-155 Reduces the Expression of CXCR5

**Fig. 4:**
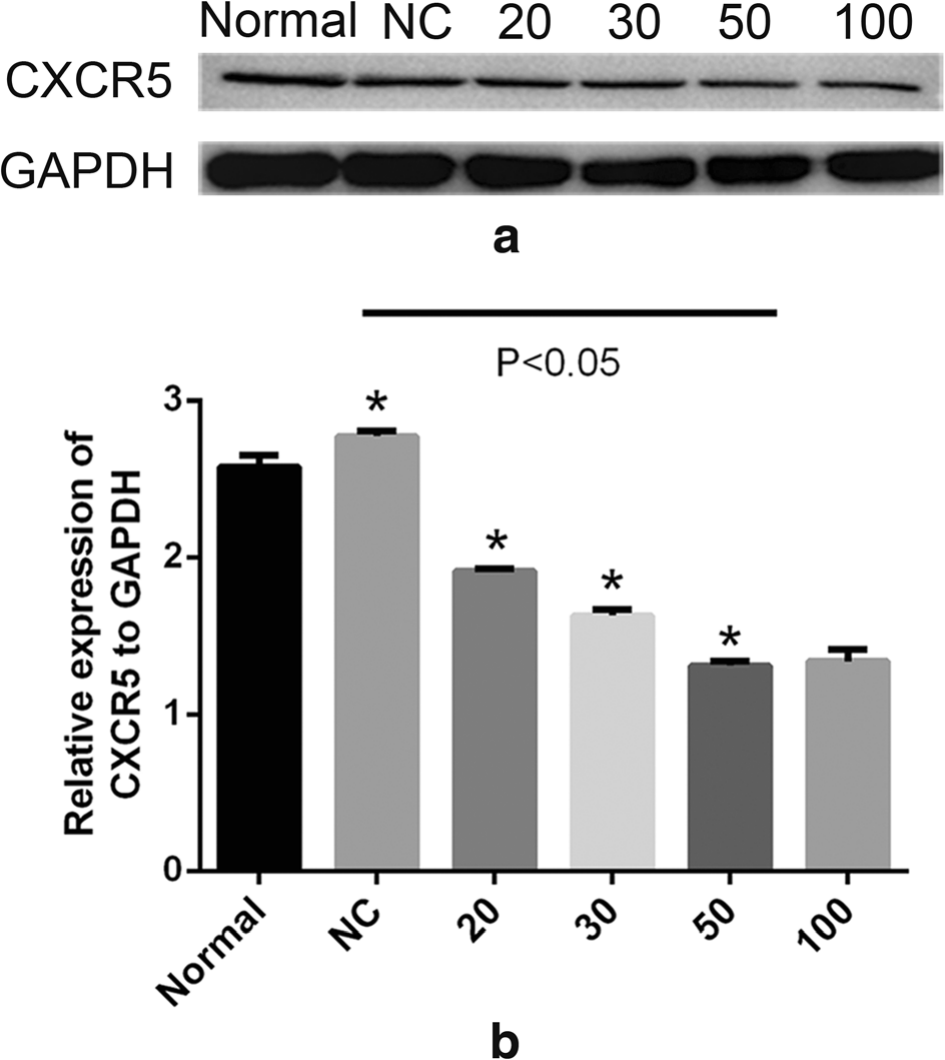
Western blot assay of the protein levels of CXCR5 in HRMCs transfected with a miR-155 mimic. (**a**) Cells were transfected with a miR-155 mimic at 20 nmol/mL, 30 nmol/mL, 50 nmol/mL, and 100 nmol/mL, and the levels of CXCR5 protein were determined. (**b**) Relative expression of CXCR5 to GAPDH was calculated. Statistical analyses were performed by ANOVA followed by Dunnett’s test. *p < 0.05.

CXCR5 is a unique receptor for CXCL13 [25]. To validate whether CXCR5 was a target gene of miR-155 in HRMCs, we transfected miR-155 mimic into HRMCs. Overexpression of miR-155 in HRMCs resulted in a significant decreased expression of CXCR5. Moreover, a concentration of 50 nmol/mL miR-155 mimic could dramatically inhibit the expression of CXCR5 (Fig. 4).

### MiR-155 Reduces ERK Tyrosine Phosphorylation

**Fig. 5:**
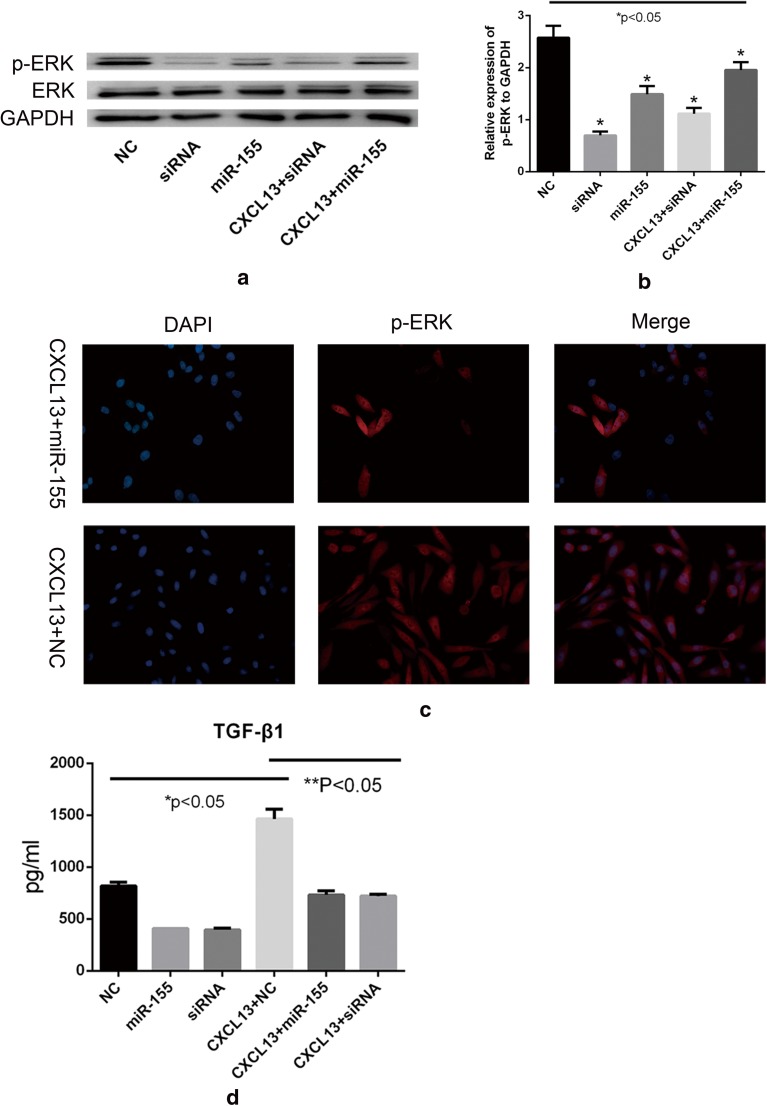
Phosphorylation of ERK in HMRCs and measurement of the concentration of TGF-β1 in supernatants of HRMCs using ELISA. (**a**–**c**) Western blot and immunofluorescent analysis in HRMCs transfected with siRNA or miR-155 mimic with or without CXCL13 treatment. (**b**) Relative expression of CXCR5 to GAPDH was calculated. (**c**) Immunofluorescent measurement of the tyrosine phosphorylation level in ERK (red). (**d**) The HRMCs were incubated for 12 h after treatment, and the supernatants were then collected for detecting TGF-β1 levels. Statistical analyses were performed by ANOVA followed by Dunnett’s test. *p < 0.05 (NC *versus* CXCL13 + NC, miR-155, or siRNA); **p < 0.05 (CXCL13 + NC *versus* CXCL13 + miR-155 or CXCL13 + siRNA).

Phosphorylation of ERK is associated with increased cell proliferation rate [29]. To investigate whether increasing the abundance of miR-155 would reduce ERK phosphorylation, we transfected the miR-155 mimic into HRMCs and then detected the level of ERK phosphorylation using western blot and immunofluorescent staining (Fig. 5a–c).

We found that CXCL13 could significantly increase the protein level of phosphorylated ERK in miR-155 mimic-transfected cells, and increased miR-155 can reduce ERK tyrosine phosphorylation (Fig. 5a, b). We also investigated whether the inhibition of p-ERK formation by miR-155 was mediated by CXCR5. We used a siRNA-specific for silencing the expression of CXCR5 and found a marked decrease in the level of p-ERK (Fig. 5a, b). The decrease in phosphorylation of ERK was similar to that obtained by increasing the abundance of miR-155. Thus, the results are consistent with miR-155 reducing the level of p-ERK by downregulating CXCR5.

### MiR-155 Suppresses TGF-β1 Production by HRMCs

TGF-β1 has the ability to stimulate mesangial cells and tubular epithelial cells to undergo myofibroblastic activation or transition *in vitro* [33]. In this study, we measured the concentration of TGF-β1, MCP-1, and IL-1 in the supernatants of HRMC culture system using ELISA. Upon treating HRMCs with CXCL13, the concentration of TGF-β1 increased. Moreover, the concentration of TGF-β1 decreased when we increased the abundance of miR-155 in HRMCs (Fig. 5d). Besides, the concentration of MCP-1 and IL-1 showed no significant change in the supernatant of CXCL13-treated HRMCs.

## DISCUSSION

MiR-155 is an important effector in immune system which could regulate innate and adaptive immune responses [9, 17]. Increasing evidence indicates that many miRNAs such as miR-146, miR-21, and miR-155 have the ability to negatively regulate the activation of inflammatory pathways in myeloid cells, suggesting they have anti-inflammatory effects [20]. Previous studies have shown that mice with miR-155 gene deficiency have functional defects in T and B lymphocytes and other immune cells [30] Dysregulated miR-155 expression can cause serious complications in the immune system. The expression of miR-155 is induced in multiple sclerosis (MS), rheumatoid arthritis (RA), and Sjögren’s syndrome [28]. In contrast, in SLE patients, serum miR-155 levels were found to be decreased compared with that in healthy controls and positively correlated with estimated glomerular filtration rate (eGFR) [32]. Up to date, very few studies focused on the role of miR-155 in inflammatory responses in HRMCs. Mesangial cells, endothelial cells, and podocytes are three main cell types in the glomerulus [35]. Mesangial cells and their matrix form the central stalk of the glomerulus and interact closely with endothelial cells and podocytes [14]. Aberrant proliferation, apoptosis, and activation of mesangial cells are frequently observed in LN [27]. In this study, we found that CXCL13 increased HRMCs proliferation, while overexpression of miR-155 could abrogate the CXCL13-stimulated cell proliferation.

MiRNA-mediated gene regulation usually reduces the amount of target proteins [22]. To find the target of miR-155, we transfected miR-155 mimic into HRMCs and found the decreased levels of CXCR5. MiR-155 is a component of the inflammatory response and regulates the ERK-MAPK signaling pathway in T cells [24]. However, thus far there has been no information regarding the relationship between ERK and miR-155 in HRMCs. P-ERK can phosphorylate a wide range of cellular substrate, modulates the transcriptional activity of the cell, and triggers cell growth and differentiation [13, 21]. We transfected a miR-155 mimic or siRNA in HRMCs and found decreased phosphorylation of ERK and inhibited cell proliferation rate. These results suggest that the decrease in p-ERK formation was due to the inhibition of CXCR5. But CXCR5 expression was only partially suppressed in miR-155 mimic-transfected HRMCs, suggesting that there may be an alternative pathway independent of miR-155 and CXCR5.

TGF-β1 belongs to a family of cytokines involved in many physiological processes, including growth, differentiation, proliferation, tissue remodeling, and wound healing [8]. In LN, TGF-β1 induces mesangial cells to undergo myofibroblastic activation or transition. When we treated HRMCs with CXCL13, the concentration of TGF-β1 increased; conversely, when we transfected the miR-155 mimic into HRMCs, the level of TGF-β1 decreased. These results indicated that CXCL13 may promote myofibroblastic activation of HRMCs, but miR-155 mimic abrogates this process.

In summary, our study has shown that miR-155 can reduce the proliferation of HRMCs and the production of TGF-β1 by downregulating the expression of the CXCR5-ERK signaling pathway upon CXCL13 stimulation. Our findings suggest that miR-155 is involved in the disease pathogenesis of LN and may be further validated as a new therapeutic target for treating LN.
